# Emotion expressions shape human social norms and reputations

**DOI:** 10.1016/j.isci.2021.102141

**Published:** 2021-02-04

**Authors:** Celso M. de Melo, Kazunori Terada, Francisco C. Santos

**Affiliations:** 1 CCDC U.S. Army Research Laboratory, Playa Vista, CA 90094, USA; 2 Gifu University, 1-1 Yanagido, Gifu 501-1193, Japan; 3 INESC-ID and Instituto Superior Técnico, Universidade de Lisboa, IST-Taguspark, 2744-016 Porto Salvo, Portugal

**Keywords:** Psychology, Research Methodology Social Sciences, Social Sciences

## Abstract

The emergence of pro-social behaviors remains a key open challenge across disciplines. In this context, there is growing evidence that expressing emotions may foster human cooperation. However, it remains unclear how emotions shape individual choices and interact with other cooperation mechanisms. Here, we provide a comprehensive experimental analysis of the interplay of emotion expressions with two important mechanisms: direct and indirect reciprocity. We show that cooperation in an iterated prisoner's dilemma emerges from the combination of the opponent's initial reputation, past behaviors, and emotion expressions. Moreover, all factors influenced the social norm adopted when assessing the action of others — i.e., how their counterparts' reputations are updated – thus, reflecting longer-term consequences. We expose a new class of emotion-based social norms, where emotions are used to forgive those that defect but also punish those that cooperate. These findings emphasize the importance of emotion expressions in fostering, directly and indirectly, cooperation in society.

## Highlights

• Emotion expressions' role in shaping reputation in society has been neglected
• In our experiment, others had positive/negative reputation, strategy, and emotion
• Results showed that emotion shaped how participants assigned reputations to others
• This exposes a new class of emotion-based social norms missing in the literature

## Graphical Abstract

**Figure:**
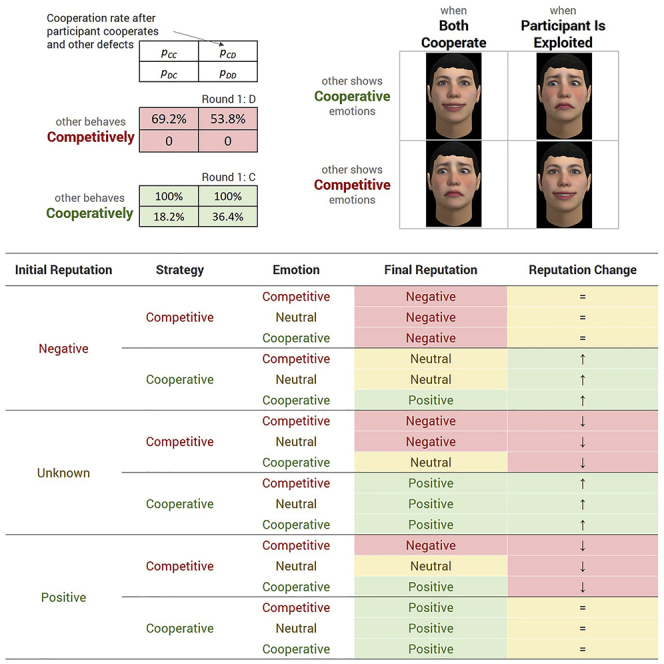

## Introduction

The evolution of cooperation among humans often relies on reputations assessing strangers' past behavior (Alexander, 1987; Nowak and Sigmund, 1998, 2005; Ohtsuki and Iwasa, 2004; Milinski, 2016). Prior research identified social norms for updating these reputations, a set of rules defining which actions are perceived as good or bad actions (Bicchieri, 2005; Brandt and Sigmund, 2004, 2005; 2005; Hitoshi et al., 2020; Leimar and Hammerstein, 2001; Ohtsuki and Iwasa, 2004, 2006, 2007; Kandori, 1992; Milinski et al., 2001; Nowak and Sigmund, 1998, 2005; Pacheco et al., 2006; Panchanathan and Boyd, 2003; Santos et al., 2018; Sasaki and Okada, 2017). This research has, however, neglected the effects of nonverbal signals on these social norms. Building on a growing literature showing effects of emotion expressions on decision-making (de Melo and Terada, 2019, 2020; de Melo et al., 2014; Frank, 2004; Keltner and Lerner, 2010; Lerner et al., 2015; Scherer and Moors, 2019; van Kleef et al., 2010), here we show that others' emotion expressions during repeated interaction play a critical role in determining how others' reputations are updated. We present an experiment where participants (*n* = 711) engaged in the iterated prisoner's dilemma with counterparts that had positive, unknown, or negative reputation, acted cooperatively or competitively (Hilbe et al., 2013; Press and Dyson, 2012; Stewart and Plotkin, 2013), and showed positive, neutral, or negative emotion expressions (de Melo and Terada, 2019; de Melo et al., 2014). The experimental results revealed clear effects of initial reputation, behavior, and emotion expression on cooperation and final reputation. These emotion-based social norms emphasize the insufficiency of others' reputations and actions in explaining how reputation is updated and show that nonverbal communication shapes how reputation is built in society.

There is increasing evidence that emotion expressions can influence human decision-making (de Melo and Terada, 2019, 2020; de Melo et al., 2014; Frank, 2004; Keltner and Lerner, 2010; Lerner et al., 2015; Scherer and Moors, 2019; van Kleef et al., 2010). Recent studies show that emotion displays can enhance or hinder cooperation, according to the contextual meaning of the expressions (de Melo and Terada, 2019, 2020; de Melo et al., 2014; van Kleef et al., 2010). This experimental evidence aligns with general arguments that emotion expressions serve important social functions, including communicating one's mental states to others (Keltner and Haidt, 1999; Keltner and Lerner, 2010; Morris and Keltner, 2000). Moreover, it is, in general, accepted that emotions are elicited from, conscious or nonconscious, appraisal of ongoing events with respect to the individual's goals (Frijda, 1986; Scherer and Moors, 2019). Thus, different emotions can be experienced as a result of different appraisal patterns which, then, result in concomitant physiological experiences, action tendencies, and expressions. Expressed emotions, therefore, reflect differentiated information about how others are appraising the ongoing interaction with respect to their goals (de Melo et al., 2014; Hareli and Hess, 2010; van Kleef et al., 2010). Accordingly, experimental researchers have shown that people are able to make inferences about others' goals from their emotion expressions (Hareli and Hess, 2010; Parkinson and Simons, 2009), including in decision-making tasks (de Melo and Terada, 2019, 2020; de Melo et al., 2014; van Kleef et al., 2004, 2010). However, whereas this work, including our own, has focused on the consequences of others' emotion expressions on the receiver's immediate responses, the impact of these nonverbal cues on the expresser's reputation – thus reflecting longer-term consequences – has been left unexplored, let alone its potential role in the evolution of human cooperation.

Reciprocity, in several of its flavors, remains as one of the most fundamental cooperation principles discovered to date (Axelrod, 1984; Rand and Nowak, 2013; Sigmund, 2016; Trivers, 1971). In direct reciprocity, cooperation emerges from long-term interactions where players have the chance to return a favor at a later stage or retaliate against wrongdoers (Trivers, 1971). Famous strategies such as Tit-for-Tat, or the recently discovered zero-determinant strategies (Hilbe et al., 2013; Press and Dyson, 2012; Stewart and Plotkin, 2013), provide insightful examples of the advantages and complexities of conditional behaviors based on past interactions. Cooperation may also emerge in the absence of preceding experiences with an opponent. Humans exhibit an astounding ability to judge strangers' behavior towards others (Alexander, 1987; Bicchieri, 2005; Hitoshi et al., 2020; Leimar and Hammerstein, 2001; Ohtsuki and Iwasa, 2004; Kandori, 1992; Milinski, 2016; Nowak and Sigmund, 1998, 2005; Panchanathan and Boyd, 2003), communicate those judgments through reputation mechanisms such as gossip (Dunbar and Dunbar, 1998; Giardini and Conte, 2012; Giardini and Vilone, 2016; Sommerfeld et al., 2007), and behave with strangers based on those reputations (Milinski, 2016; Gross and De Dreu, 2019). This is indirect reciprocity at work. Cooperation with strangers can, thus, emerge in one-shot interactions if information about strangers' past behavior is available and reliable (Hilbe et al., 2018; Santos et al., 2018b; Radzvilavicius et al., 2019; Szabolcs et al., 2016; Uchida, 2010), even though the payoff-maximizing move would be to defect. When combined with particular social norms (Ohtsuki and Iwasa, 2006), defining how individuals should behave and how reputations should be updated, indirect reciprocity can effectively promote cooperation in a community.

Some experimental work indicates that these two forms of reciprocity can interact in important ways, with the effects of direct interaction often overriding the effects of reputation in time (Molleman et al., 2013). This interplay, furthermore, may conflict with humans' cognitive capacity to reason on multiple (and, possibly, conflicting) sources of information through complex heuristics (Feldman, 2000; Melamed et al., 2020; Radzvilavicius et al., 2019; Santos et al., 2018, 2018b). Also, actions and assessment of others' actions are often made in absence of complete information about the decision maker or recipient's reputations and motivation for past actions (Feldman, 2000; Radzvilavicius et al., 2019; Uchida, 2010). Here we resort to a novel experimental setup showing that nonverbal emotion signals may provide an escape hatch to this complexity. Despite the complexity of including an additional layer of information, we show that human cooperation and social norms emerge through simple rules that depend on opponent's emotion profiles, reputations, and past actions. Interestingly, even after several rounds of first-hand interactions, emotions and reputations continue to influence participants' choices. We show how expressions of emotion reveal a nuanced view of the decision maker's mind, and, in some cases, help forgive those that defect or punish those that cooperate.

**Figure 1:**
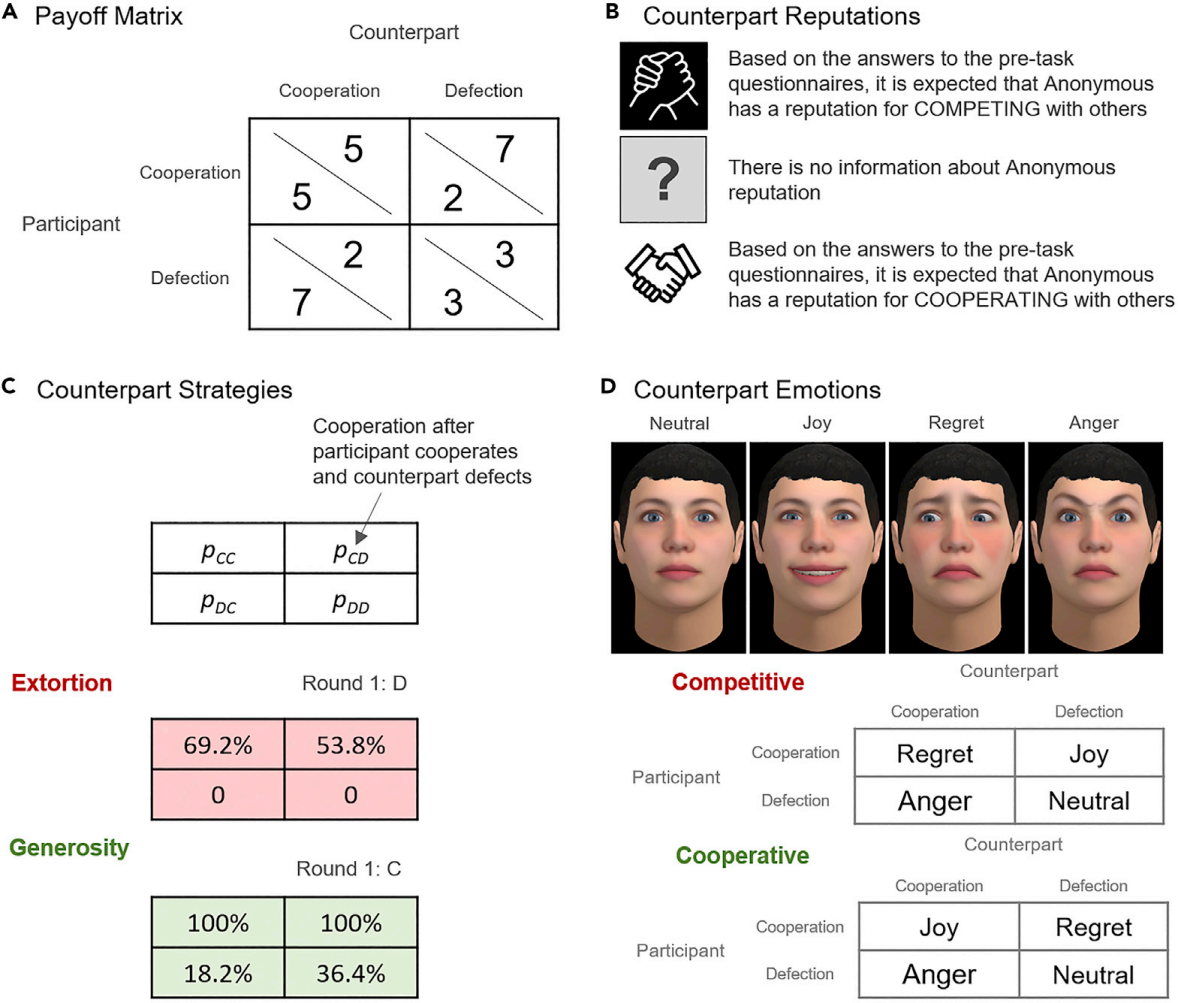
Experimental design for the iterated prisoner's dilemma task (A) The Prisoner's dilemma payoff matrix adopted in the experiments. (B) The reputation manipulation: participants were instructed that the counterpart's reputation was negative, unknown, or positive based on pre-task questionnaires. (C) The counterpart strategies: each strategy was defined by the cooperation probability following each possible outcome of the prisoner's dilemma (Hilbe et al., 2013). (D) The cooperative and competitive emotion expression patterns: the facial expressions were shown after each outcome, according to the corresponding pattern.

We present an experiment where participants face an iterated prisoner's dilemma with 20 rounds. In each round, the two players could either cooperate or defect, receiving a return given by the payoff matrix in Figure 1A. The task payoff had financial consequences for participants, as each point would increase their chances of winning a $30 lottery. Participants were given the opportunity to learn about their counterpart's reputation prior to the task, which was either negative, unknown, or positive. This reputation, according to the instructions, had presumably been calculated based on responses to questionnaires administered earlier (see Figure 1B and further details in the Supplemental information). Participants, however, were informed that their own (initial) reputation would not be shared with the counterpart – i.e., from the perspective of the counterpart, the participant's reputation was always unknown. Thus, even though prior work suggests that own reputation can influence behavior and social norms (Nowak and Sigmund, 1998, 2005; Ohtsuki and Iwasa, 2004, 2006), to keep the experimental design simple, here we simply controlled for this factor and left the study of higher order social norms (Santos et al., 2018) for future work. Furthermore, to ensure experimental control in the administration of the reputation, strategy, and emotion expression manipulations, participants always engaged with computer scripts (de Melo and Terada, 2019). All experimental procedures were approved by the Gifu University IRB, and participants were fully debriefed at the end.

Participants engaged with counterparts that either acted cooperatively or competitively. To implement this behavior, we looked at recent work on zero-determinant strategies, which include strategies that unilaterally enforce a linear relationship between the players' payoffs (Press and Dyson, 2012). On the competitive end, extortion strategies ensure that the counterpart cannot earn more than the extortionist by exploiting often while cooperating just enough to keep the counterpart cooperating (Figure 1C, top) (Hilbe et al., 2013). On the cooperative end, generous strategies reward cooperation while only punishing defection mildly (Figure 1C, bottom) (Stewart and Plotkin, 2013). See Supplemental information for details and proof that the proposed strategies and payoff matrix meet the requirements for zero-determinant strategies. Prior work (de Melo and Terada, 2020), indicates that emotion expressions moderated the effect of these strategies on cooperation; however, this work did not explore the impact of these factors on social norms. The experimental design presented here, thus, allows for the systematic study of the effects of direct reciprocity – through the counterpart's strategy – crossed with the effects of indirect reciprocity – through the counterpart's reputation.

Participants engaged in the prisoner's dilemma with counterparts that showed cooperative, neutral, or competitive emotion expressions. To accomplish this, players were represented by virtual faces, which is a methodology that has been shown in the past to have high ecological validity while allowing for high experimental control over the emotion expressions (Blascovich et al., 2002; de Melo and Terada, 2019; de Melo et al., 2014). The counterparts' virtual face was young and Caucasian and was kept constant across conditions to control for biases related to physical characteristics of the face (de Melo and Terada, 2019; Todorov et al., 2008). The face showed typical, validated, expressions (de Melo and Terada, 2019; de Melo et al., 2014) for joy, regret, and anger (Figure 1D). After the outcome of the round was revealed, the facial expression was animated in real-time and shown to the participant (see the Video S1 showing the software developed for this experiment). Building on prior work that certain patterns of emotions can promote or hinder cooperation (de Melo and Terada, 2019, 2020; de Melo et al., 2014), we implemented emotion patterns compatible with cooperative, neutral, and competitive intentions (Figure 1D): cooperative – joy following mutual cooperation, regret after exploiting the participant, anger after being exploited, and neutral otherwise; neutral – no emotion was shown; and, competitive – regret following mutual cooperation (given that it missed the opportunity to exploit the participant), joy after exploiting the participant, anger after being exploited and, neutral otherwise.

Video S1. Software used for the iterated prisoner's dilemma experiment, Related to Figure 1

## Results

**Figure 2:**
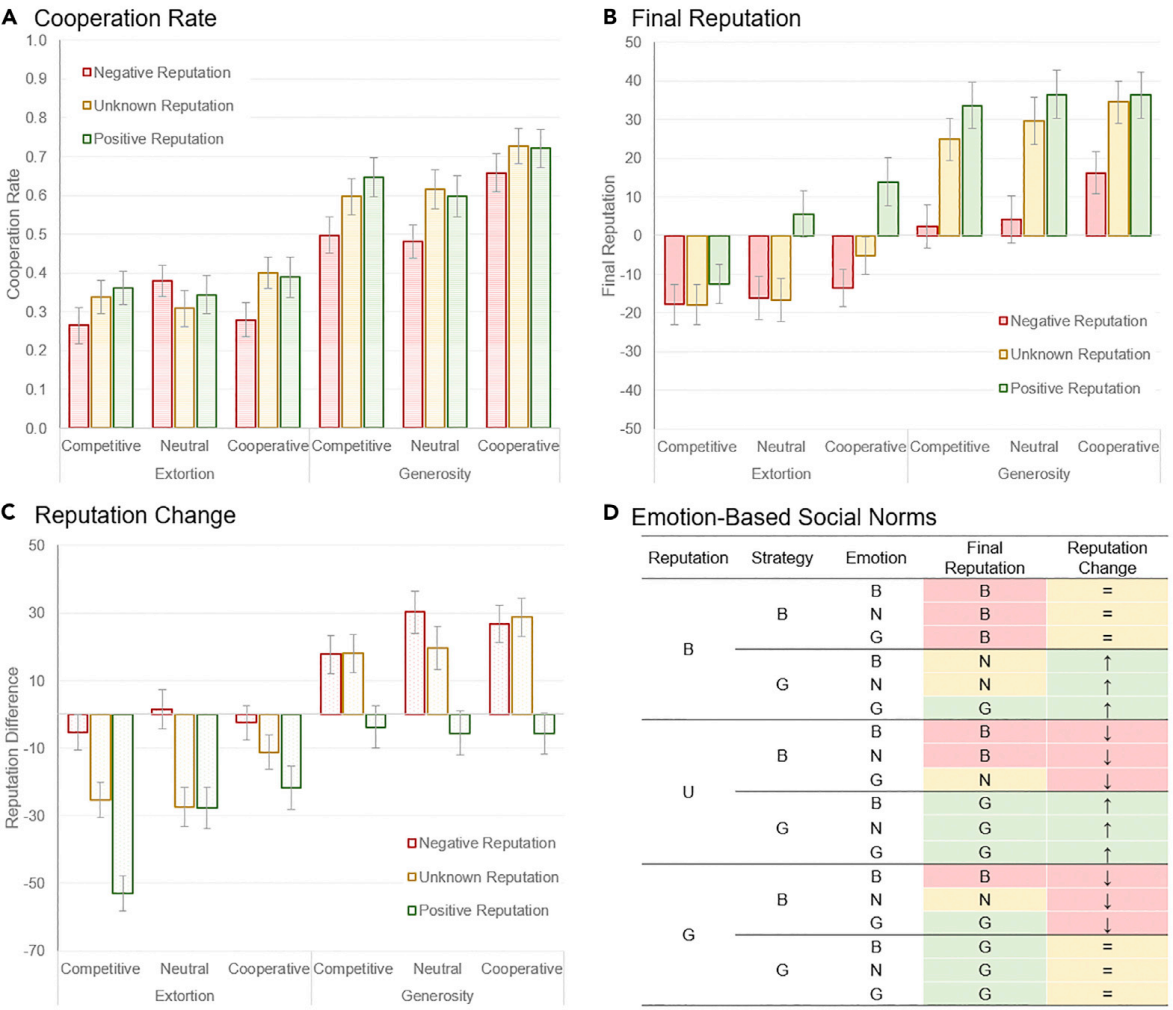
Experiment results for the iterated prisoner's dilemma task (A) Cooperation rate across the 20 rounds. Error bars correspond to standard errors. (B) Counterpart reputation perception at the end of the task. Error bars correspond to standard errors. (C) Change in counterpart reputation perception, calculated as difference between final and initial reputation. Error bars correspond to standard errors. (D) Social norms based on reputation, strategy, and emotion. Labels for counterpart reputation, strategy, and emotion: negative emotion or reputation (*bad*, B), unknown reputation (U), neutral emotion (N), and positive emotion or reputation (*good*, G). Labels and colors for final reputation perception: negative (B, red), neutral (N, yellow), and positive (G, green). Red and green cells correspond to values that are statistically significantly different than zero (see Table S1 in Supplemental information). Labels and colors for reputation change: downwards (↓, red), neutral (=, yellow), upwards (↑, green). Red and green cells correspond to values that are statistically significantly different than zero (see Table S1 in Supplemental information).

The experiment, thus, followed a 3 × 2 × 3 between-participants factorial design: reputation (negative vs. unknown vs. positive) × strategy (extortion vs. generosity) × emotion (competitive vs. neutral vs. cooperative). We first looked at cooperation rate, averaged across the 20 rounds. For an analysis of round effects, please see the Supplemental information. In the Supplemental information we also provide an extended analysis confirming that, in the first round, cooperation rate was only influenced by reputation. We ran an analysis of variance (ANOVA) on cooperation rate (Figure 2A), which showed a main effect of reputation (*F*(2, 693) = 5.65, p = 0.004, partial *η*^*2*^ = 0.016) in the entire time span of the game. Indeed, post-hoc tests with a Bonferroni correction revealed that participants cooperated less with counterparts with a negative reputation than unknown (p = 0.020) or positive (p = 0.007) reputations. This result, thus, emphasizes that the effect of indirect reciprocity still existed, despite 20 rounds of direct interaction with the counterpart (Melamed et al., 2020). Moreover, we note that participants appeared to cooperate with those with unknown reputation similarly to those with a positive reputation. There was a main effect of strategy (*F*(1, 693) = 155.51, p < 0.001, partial *η*^*2*^ = 0.183), with participants cooperating more with generous than extortion strategies. This effect emphasizes the strength of direct reciprocity on overall cooperation (de Melo and Terada, 2020; Molleman et al., 2013). There was also a main effect of emotion (*F*(2, 693) = 5.35, p = 0.005, partial *η*^*2*^ = 0.015), with participants cooperating more with players showing cooperative emotions than neutral (p = 0.019) or competitive (p = 0.011) emotions. This effect is in line with earlier research on cooperation and emotion expressions (de Melo and Terada, 2019, 2020; de Melo et al., 2014), despite the existence of direct and third-party information (reputations) available on the counterpart.

To understand the combined effects of actions, current reputation and emotion on the social norm that reckons the next reputation of an individual, we asked participants to rate, on a 100-point Likert scale (−50, *likely to compete*, to 50, *likely to cooperate*), the counterpart's reputation at the start and end of the task (see Supplemental information for details). The analysis focused on perceived reputation at the end, and reputation change (final reputation minus initial reputation) – see Supplemental information for an extended analysis confirming that perceived reputation at the start was only influenced by counterpart's reputation. We first ran an ANOVA on final reputation. This analysis revealed a main effect of (initial) reputation (Figure 2B, *F*(2, 693) = 24.69, p < 0.001, partial *η*^*2*^ = 0.067). Post-hoc tests revealed that final reputation was higher for those with a positive reputation than unknown reputation (p = 0.004) and higher for those with unknown reputation than negative reputation (p < 0.001). This result reveals that the current reputation of an individual was taken into consideration when being evaluated by others, suggesting the use of a high-order social norm (Nowak and Sigmund, 1998; Ohtsuki and Iwasa, 2006) where the actions of players do not suffice to assess which actions are deemed good or bad. There was also an effect of the strategy on the final reputation (*F*(1, 693) = 157.09, p < 0.001, partial *η*^*2*^ = 0.185): Counterparts with generous strategies received a higher final reputation than those with extortion strategies. Emotions also played an important, yet subtler role (*F*(2, 693) = 6.52, p = 0.002, partial *η*^*2*^ = 0.018): Those showing cooperative emotion received higher final reputation than those showing competitive displays (p = 0.001). The analysis also revealed a reputation × strategy interaction (*F*(2, 693) = 4.81, p = 0.008, partial *η*^*2*^ = 0.014), with unknown reputation being influenced the most by strategy, when compared to negative or positive reputations.

An ANOVA on reputation change (Figure 2C) showed an effect of reputation (*F*(2, 693) = 42.43, p < 0.001, partial *η*^*2*^ = 0.109), with participants correcting their reputation ratings, when averaging across all conditions, downwards for positive reputations and upwards for negative reputations. This effect emphasizes a synergy between direct experience and initial expectations derived from the counterpart's reputation (Melamed et al., 2020; Molleman et al., 2013). There was an effect of strategy (*F*(1, 693) = 148.45, p < 0.001, partial *η*^*2*^ = 0.176), with reputations moving downwards for extortionists and upwards for generosity. There was also an effect of emotion (*F*(2, 693) = 5.70, p = 0.004, partial *η*^*2*^ = 0.016), with reputations being lowered when competitive emotion was shown, and raised when cooperative emotion was expressed. There was a reputation × strategy interaction (*F*(2, 693) = 3.73, p = 0.025, partial *η*^*2*^ = 0.011), with unknown reputations once again being impacted the most by strategy.

These effects, thus, reveal that initial reputation, strategy, and emotion expressions, all contributed to shape the counterpart's reputation. To gain further insight, we ran one-way *t* tests, for each experimental condition, to understand if final reputation and reputation change were impacted in a meaningful way – i.e., if the value was statistically significantly different than zero (see Table S1 in Supplemental information for details). Figure 2D summarizes all effects. The first three columns correspond to the different reputations (*G* and *B* indicate good/positive and bad/negative, respectively, and *U* unknown), strategy (*G* and *B* stands for generous and extortion strategies, respectively), and emotion (*G* and *B* stands for cooperative and competitive emotions, respectively). In the “Final Reputation” column, colors encode values that were statistically significant (red for negative, green for positive) and non-significant (yellow for neutral) reputations. Similarly, the “Reputation Change” column, color-codes statistically significant (red for upwards, green for downwards) and non-significant (yellow for neutral) changes in reputation perceptions.

## Discussion

The results summarized in Figure 2D reveal a surprisingly simple social norm combining the effects of reputations, actions and emotions, all providing resourceful information to the evaluator. In particular, emotions offer a confirmation mechanism, both for forgiveness and punishment. First, emotions help to forgive those that defect while having a positive (good) reputation; in other words, defectors (in this case, extortionists) with a good reputation will keep their status as long as they display a cooperative emotion. The key concept “justified defection” in social norms of indirect reciprocity – whereby one should refuse to cooperate with those having a negative reputation (Panchanathan and Boyd, 2003; Ohtsuki and Iwasa 2006; Sigmund 2016; Yamamoto et al., 2020) – can therefore be revisited through the eyes of emotional expressions. Moreover, emotions show the potential to be used as an efficient error-correction mechanism, a feature of central importance in the evolutionary dynamics of cooperation. Second, to recover from a bad reputation, being generous was not sufficient: Good intentions had to be confirmed by cooperative emotions, providing another device for correcting misevaluations, in this case, whenever an individual is rehabilitated from a “bad” to a “good” status.

Emotion theorists have long recognized that the origins of human emotions are inherently social (Frijda, 1986; Haidt, 2003; Scherer and Moors, 2019). Though emotions emerge from appraisal of events with respect to the individual's beliefs and goals, these appraisals often pertain to events and decisions impacting others. The ability to empathize with the fate of others has been argued to be at the origin of human systems of moral judgment and communication (Alexander, 1987; Bicchieri, 2005; Dunbar and Dunbar, 1998; Giardini and Conte, 2012; Giardini and Vilone, 2016; Gross and De Dreu, 2019; Hitoshi et al., 2020; Leimar and Hammerstein, 2001; Ohtsuki and Iwasa, 2004; Kandori, 1992; Milinski, 2016; Nowak and Sigmund, 1998, 2005; Panchanathan and Boyd, 2003; Radzvilavicius et al., 2019; Sommerfeld et al., 2007; Traag et al., 2011). Here we show that emotion expressions are an intrinsic component of these complex systems defining social norms prescribing reputations to others and helping sustain cooperation through direct and indirect reciprocity. This work emphasizes the insufficiency of reputation priors and actions in determining whether an individual is worthy of cooperation, now and in future interactions with other members of the community. Furthermore, research exposes the difficulty in sustaining cooperation in the presence of private, noisy, and incomplete reputation systems (Feldman, 2000). Emotion expressions fill this gap by helping disambiguate social situations and supporting real-time inferences about others' intentions and social norms. Our results clearly show that reputation and behavior cannot code, by themselves, the social norms adopted by participants in the experiment (see Figure 2D), a result of particular relevance for evolutionary biologists addressing the evolution of cooperation through indirect reciprocity, and its interplay with direct reciprocity. Only emotion expression, for instance, explains why an individual that starts with a negative reputation but behaves cooperatively and expresses cooperative emotion can end with a positive reputation. This work, thus, exposes a whole new class of emotion-based social norms that has been missing and potentially complements previous work on social norms of different orders (Alexander, 1987; Bicchieri, 2005; Feldman, 2000; Hitoshi et al., 2020; Leimar and Hammerstein, 2001; Kandori, 1992; Milinski, 2016; Nowak and Sigmund, 1998, 2005; Ohtsuki and Iwasa, 2004; Panchanathan and Boyd, 2003). At the same time, our work also confirms the impressive role of indirect reciprocity and third-party information in human cooperation. Reputations are shown to still have an impact on participants' decisions, even after a significant number of first-hand interactions, and potentially puzzling messages provided by emotions.

Finally, this work has important practical implications, in particular, for the design of autonomous machines – such as robots, autonomous vehicles, and personal assistants (de Melo et al., 2019; Stone and Lavine, 2014). As these machines become increasingly pervasive, their success hinges on humans being willing to cooperate with them (de Melo and Terada, 2019), or on how machines may trigger cooperation among humans (Crandall et al., 2018). Our results suggest that designers should not only consider reputation mechanisms (e.g., similar to online trading systems) and appropriate behavior (e.g., generous or tit-for-tat strategies), but use nonverbal communication, such as emotion expressions, to build trust and encourage cooperation with humans. Because they can be designed from the ground up, moreover, these machines are in a unique position to shape human behavior and promote cooperation in society.

### Limitations of the study

The study presented here has some limitations that introduce opportunities for future work. The experimental design controlled for the effect of the participants' reputation on their behavior by always assigning them an unknown reputation. However, prior work suggests that own reputation can influence behavior and social norms (Nowak and Sigmund, 1998, 2005; Ohtsuki and Iwasa, 2004, 2006) and, thus, it is worth exploring if this factor interacts in important ways with other's emotion expressions, reputation, and strategy. In this work, we also controlled for possible biases introduced by the counterpart's physical characteristics by keeping the virtual face constant across conditions but, prior research suggests that people can form important judgments from these characteristics (de Melo and Terada, 2019; Todorov et al., 2008) and, so, future work should also account for the effect of this factor on direct and indirect reciprocity. Our sample was collected from a single online pool (Mechanical Turk) and, therefore, we cannot exclude the possibility of a shared sense of social group membership between participants and their counterparts (e.g., participants tended to be as cooperative with counterparts with unknown and positive reputations). However, participants may behave differently and follow different social norms with out-group members. It is, thus, important to study the role of emotion expressions in reputation building with different samples, including involving participants from different social groups. Finally, individual factors can influence people's propensity for cooperation (Balliet et al., 2009; Eckel and Grossman, 1999) and should also be explored, in future work, in conjunction with the factors studied here.

### Resource availability

#### Lead contact

Further information and requests for resources should be directed to and will be fulfilled by the lead contact, Celso M. de Melo (celso.miguel.de.melo@gmail.com).

#### Materials availability

This study did not generate new unique reagents.

#### Data and code availability

The published article includes all experimental data collected and analyzed during this study with the supplemental materials. The code supporting the current study has not been deposited in a public repository because it includes proprietary and licensed software but some materials are available from the corresponding author on request.

## Ethics declaration

All experimental methods were approved by the Medical Review Board of Gifu University Graduate School of Medicine (IRB ID#2018-159). As recommended by the IRB, written informed consent was provided by choosing one of two options in the online form: 1) “I am indicating that I have read the information in the instructions for participating in this research and have had a chance to ask any questions I have about the study. I consent to participate in this research.”, or 2) “I do not consent to participate in this research.” All participants gave informed consent and, at the end, were debriefed about the experimental procedures. All the experiment protocols involving human-subjects was in accordance to guidelines of the Declaration of Helsinki.

## Methods

All methods can be found in the accompanying Transparent Methods supplemental file.

## Supplemental information

Document S1. Transparent methods, Figures S1 and S2, and Table S1

Data S1. Experimental data for the iterated prisoner's dilemma experiment, Related to Figure 2

## Footnotes

Supplemental Information can be found online at https://doi.org/10.1016/j.isci.2021.102141.
