# Supplementary material for: Emotion expressions shape human social norms and reputations
Source: iScience. 2021 Feb 4;24(3):102141. doi: 10.1016/j.isci.2021.102141 (PMC7898177; doi:10.1016/j.isci.2021.102141)
Supplement: Document S1. Transparent methods, Figures S1 and S2, and Table S1 [file mmc1.pdf]

iScience, Volume *24*

Supplemental Information

Emotion expressions shape human social norms and reputations

Celso M. de Melo, Kazunori Terada, and Francisco C. Santos

Supporting Information for

Emotion expressions shape human social norms and reputations

Celso M. de Melo, Kazunori Terada, Francisco C. Santos.

Correspondence to: celso.miguel.de.melo@gmail.com

This PDF file includes:

Supplementary Text
Fig. S1-S2
Table S1
Caption for Movie S1

Other Supplementary Materials for this manuscript include the following:

Movie S1
Data S1

Transparent Methods

Participant Sample. Participants for the experiment were recruited from Amazon Mechanical Turk. All participants were from the United States and had an approval rate, based on prior work in this pool, of at least 95%. We excluded participants from prior emotion expression studies[1, 2]. To estimate the sample size for each experiment, we followed the power calculations proposed by Jacob Cohen and implemented in G*Power[3]. We estimated sample size for a 3 × 2 × 3 mixed factorial design: reputation (negative vs. unknown vs. positive) × strategy (extortion vs. generosity) × emotion (competitive vs. neutral vs. cooperative). For a small effect size (Cohen's $f = 0.15$), $\alpha = .05$, and statistical power of 0.90, the recommended total sample size was 690 participants, which rounds up to 702 participants to keep the distribution even across cells. When recruiting from this pool, it is common for some participants to fail to successfully complete the task or otherwise make data entry errors. To account for that, we increased the target sample size experiment to 720 participants. In practice, we ended up with a sample of 711 participants with the following demographics: 62.2% males; age distribution (18 to 21 years, 2.7%; 22 to 34 years, 48.5%; 35 to 44 years, 25.2%; 45 to 54 years, 13.4%; 55 to 64 years, 7.9%; over 64 years, 2.4%).

Full Anonymity. All experiments were fully anonymous for participants. To accomplish this, counterparts had anonymous names, and we never collected any information that could identify participants. To ensure understanding, participants were instructed and quizzed on these anonymity conditions prior to starting the task. To preserve anonymity with respect to experimenters, we relied on the anonymity system available through Mechanical Turk. When interacting with participants, researchers are unable to identify the participants, unless we explicitly ask for information that can identify them (e.g., name, email, or photo), which we did not. Note, however, that even though it is not possible to identify the participants, the system supports: (1) rewarding participants, which we only used to pay the lottery winner; and, (2) block participants from participating in (our) future studies, which we never used.

Financial Incentives. Participants were paid $2.50 for participating in the experiments, which is a typical amount for this online pool. Moreover, they could earn more money according to their performance in the task. Each point earned in the task was automatically converted to a ticket for a lottery worth $30.00.

Pre- and post-task questionnaires and additional task measures. Prior to receiving the instructions for the iterated prisoner's dilemma task, participants were asked to answer demographics questions – gender and age – and the 6-item slider social value orientation (SVO) scale. The SVO scale is used to measure an individual's propensity for cooperation. The administration of this scale, thus, supported the cover story that "based on the answers to pre-task questionnaires" the counterpart's reputation was negative, unknown, or positive. Participants, then, received detailed instructions for the prisoner's dilemma task, including a quiz and tutorial. Prior to starting the task, to measure initial perception of the counterpart's reputation, we asked on a 100-point Likert scale (-50, *likely to compete*, to 50, *likely to*

---

[1] de Melo C., Terada K. 2019 Cooperation with autonomous machines through culture and emotion. *PLOS ONE*, https://doi.org/10.1371/journal.pone.0224758.
[2] de Melo C., Terada K. 2020 The interplay of emotion expressions and strategy in promoting cooperation in the iterated prisoner's dilemma. *Sci. Rep.* ***10***.
[3] https://www.psychologie.hhu.de/arbeitsgruppen/allgemeine-psychologie-und-arbeitspsychologie/gpower.html (Last accessed: May-31, 2020)

cooperate): "What is *Anonymous* reputation?". After completing the task, to measure final perception of the counterpart's reputation, we asked: "Now that the task is over, what is *Anonymous* reputation?". In addition to collecting the participants' decisions in the prisoner's dilemma for each round, based on prior work[1,2,4], we collected three additional measures: (a) participants' decision time; (b) participants' self-reported emotion – from neutral, joy, sadness, anger, and regret – after each round; (c) participants' expectations of cooperation for the next round. However, for the work presented in this paper, these measures were not used.

Supplementary Text

Zero-determinant requirements for extortion and generosity strategies

Zero-Determinant (ZD) strategies are memory-one strategies in which the decision for the current round only depends on the outcome of the previous round and they enforce a linear relationship between the players' payoffs in the prisoner's dilemma (Press & Dyson, 2012). ZD strategies are written as a 5-tuple $(p_0, p_R, p_S, p_T, p_P)$, where p_0 is the player's probability of cooperation in the first round $(m = 1)$, p_i is the probability of cooperation in round $m \geq 2$ given the payoff $i \in \{R, S, T, P\}$ in the previous round. Payoff R and S are given to both players when both player cooperate and defect, respectively. If one player cooperates and the other defects, T is given to the defector and S is given to the cooperator. The relation $T > R > P > S$ is typically assumed to hold. According to Hilbe et al.[5], the probabilities of cooperation are defined as follows:

$$p_R = 1 - \phi(1 - s)(R - l) \quad (1)$$

$$p_S = 1 - \phi[(1 - s)(S - l) + T - S] \quad (2)$$

$$p_T = \phi[(1 - s)(l - T) + T - S] \quad (3)$$

$$p_P = \phi(1 - s)(l - P) \quad (4)$$

, where l, s, and ϕ are constants.

While ZD strategies are able to enforce a linear relationship between average payoff π of the ZD strategist and the expected payoff $\tilde{\pi}$ of the counterpart when the game is repeatedly and infinitely played, Hilbe et al. (*15*) showed that when the game is played M rounds, the relationship between π and $\tilde{\pi}$ follows these inequalities:

$$-\frac{p_0}{\phi M} \leq (1 - s)l + s\pi - \tilde{\pi} \leq \frac{1 - p_0}{\phi M} \quad (5)$$

.

We used the payoff values $T = 7$, $R = 5$, $P = 3$, $S = 2$, and a total number of rounds $M = 20$. The following are the values in our experiment for the constants in Equations (1)-(4), and the relation between π and $\tilde{\pi}$ predicted by the inequalities in (5):

Extortion

$l = P, s = {}^{1}/_{3}, \phi = {}^{3}/_{13}$

---

[4] de Melo C., Carnevale P., Read S., Gratch J. 2014 Reading people's minds from emotion expressions in interdependent decision making. *J. Pers. Soc. Psychol.* ***106***, 73-88.

[5] Hilbe C., Nowak M., Sigmund K. 2013 Evolution of extortion in Iterated Prisoner's Dilemma games. *Proc. Natl. Acad. Sci. U.S.A.* ***110***, 6913-6918.

$$p_0 = 0.000, p_R = 0.692, p_S = 0.000, p_T = 0.538, p_P = 0.000$$
$$\frac{1}{3} \cdot \pi + \frac{2}{3} \cdot 3 - \frac{13}{60} \leq \tilde{\pi} \leq \frac{1}{2} \cdot \pi + \frac{2}{3} \cdot 3$$

Generosity

$l = R, s = {}^1/_3, \phi = {}^3/_{11}$

$$p_0 = 1.000, p_R = 1.000, p_S = 0.182, p_T = 1.000, p_P = 0.364$$
$$\frac{1}{3} \cdot \pi + \frac{2}{3} \cdot 5 \leq \tilde{\pi} \leq \frac{1}{2} \cdot \pi + \frac{2}{3} \cdot 5 + \frac{11}{60}$$

We conducted computer simulations to confirm that the strategies used in our experiment met the zero-determinant requirements. Fig. S1 (left panel) shows that average payoffs π and $\tilde{\pi}$ are distributed within the range of the linear relationship given by the inequities in (5). Figure S1 (right panel) shows the comparison of experimental results to theoretical predictions, confirming that the relationship between the payoffs of the ZD strategist and the participants fits the linear relationship prediction.

Cooperation in first round

We ran an analysis of variance (ANOVA) on cooperation in the first round. This analysis confirmed an effect of reputation ($F(2, 693) = 6.23$, $p = 0.002$, partial $\eta^2 = 0.018$), with participants cooperating less with counterparts with a negative reputation than unknown ($p = 0.008$) or positive ($p = 0.007$) reputations. The results also suggest that participants appear to cooperate with those with unknown reputation similarly to those with a positive reputation, a comforting and timely message. As expected, however, there was no effect of strategy ($F(1, 693) = 0.235$, $p = 0.628$) and emotion ($F(2, 693) = 2.42$, $p = 0.090$). There were also no statistically significant interactions.

Round effects for cooperation rate

To understand if there were any round effects, we ran a round × reputation × strategy × emotion mixed ANOVA. The results, shown in Fig. S2, confirmed main effects for reputation ($F(2, 693) = 5.65$, $p = 0.004$, partial $\eta^2 = 0.016$), strategy ($F(1, 693) = 155.51$, $p < 0.001$, partial $\eta^2 = 0.183$), and emotion ($F(2, 693) = 5.35$, $p = 0.005$, partial $\eta^2 = 0.015$), as detailed in the main text. They also showed a main effect of round, $F(19, 13167) = 12.52$, $p < 0.001$, partial $\eta^2 = 0.018$, with cooperation tending to decrease as the game progressed – this was particularly evident in the last round, which is in line with prior work suggesting participants defect in the last round since there is no further opportunity for retribution[6]. There were no round × reputation ($F(38, 13167) = 0.69$, $p = 0.925$) and round × emotion ($F(38, 13167) = 1.08$, $p = 0.342$) interactions; however, there was a round × strategy ($F(19, 693) = 6.71$, $p < 0.001$, partial $\eta^2 = 0.010$) interaction, with cooperation tending to decrease in time for extortion but not generosity.

Reputation perceptions before the first round

To understand initial perceptions of reputation, we ran an ANOVA on the pre-task question on counterpart reputation perceptions. This analysis revealed a main effect of reputation ($F(2, 693) = 202.98$, $p < 0.001$, partial $\eta^2 = 0.369$), with negative reputations being rated lower than

[6] Kollock P. 1998 Social dilemmas: The anatomy of cooperation. *Annu. Rev. Sociol.* ***24***, 183-214.

unknown reputations ($p < 0.001$), and unknown reputations being rated lower than positive reputations ($p < 0.001$). As expected, there was no effect of strategy ($F(1, 693) = 0.001$, $p = 0.980$) and emotion ($F(2, 693) = 0.48$, $p = 0.622$). There were also no statistically significant interactions.

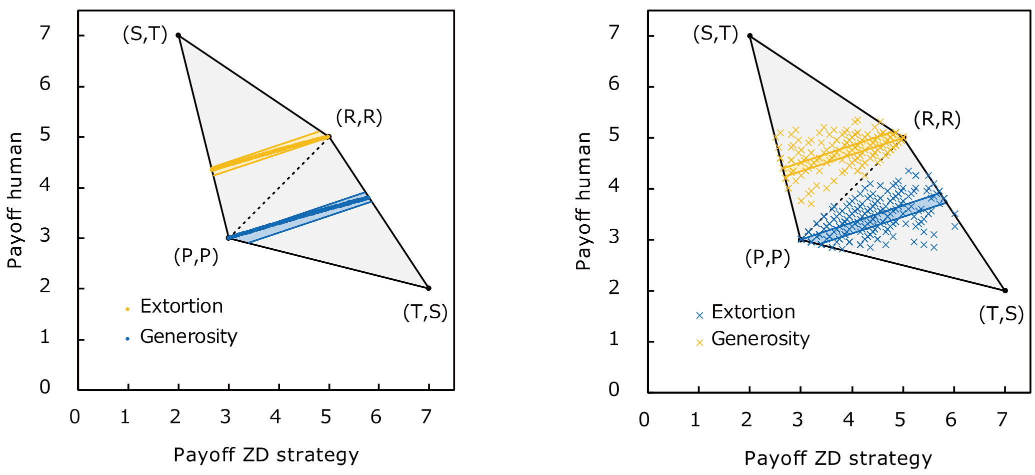

Figure S1. Experimental results, theoretical predictions, and simulations for extortion and generosity strategies, Related to Figure 1. A gray-shaded rectangular area surrounded by a solid line indicates the space of possible payoffs for the two players of the prisoner's dilemma. X-axis and y-axis indicate payoffs of ZD strategy and counterpart, respectively. The color-shaded areas between two straight colored solid lines indicate expected payoff ranges according to the inequalities in Eq. (5) — i.e., the theoretical predictions. The left panel shows a comparison of simulated payoffs to the theoretical prediction. Each dot between two color solid lines indicates the average payoff obtained from 10^3 simulated prisoner's dilemma interactions for a fixed cooperation rate (randomly chosen from 0 to 1). The right panel shows a comparison of experimental results to theoretical predictions.

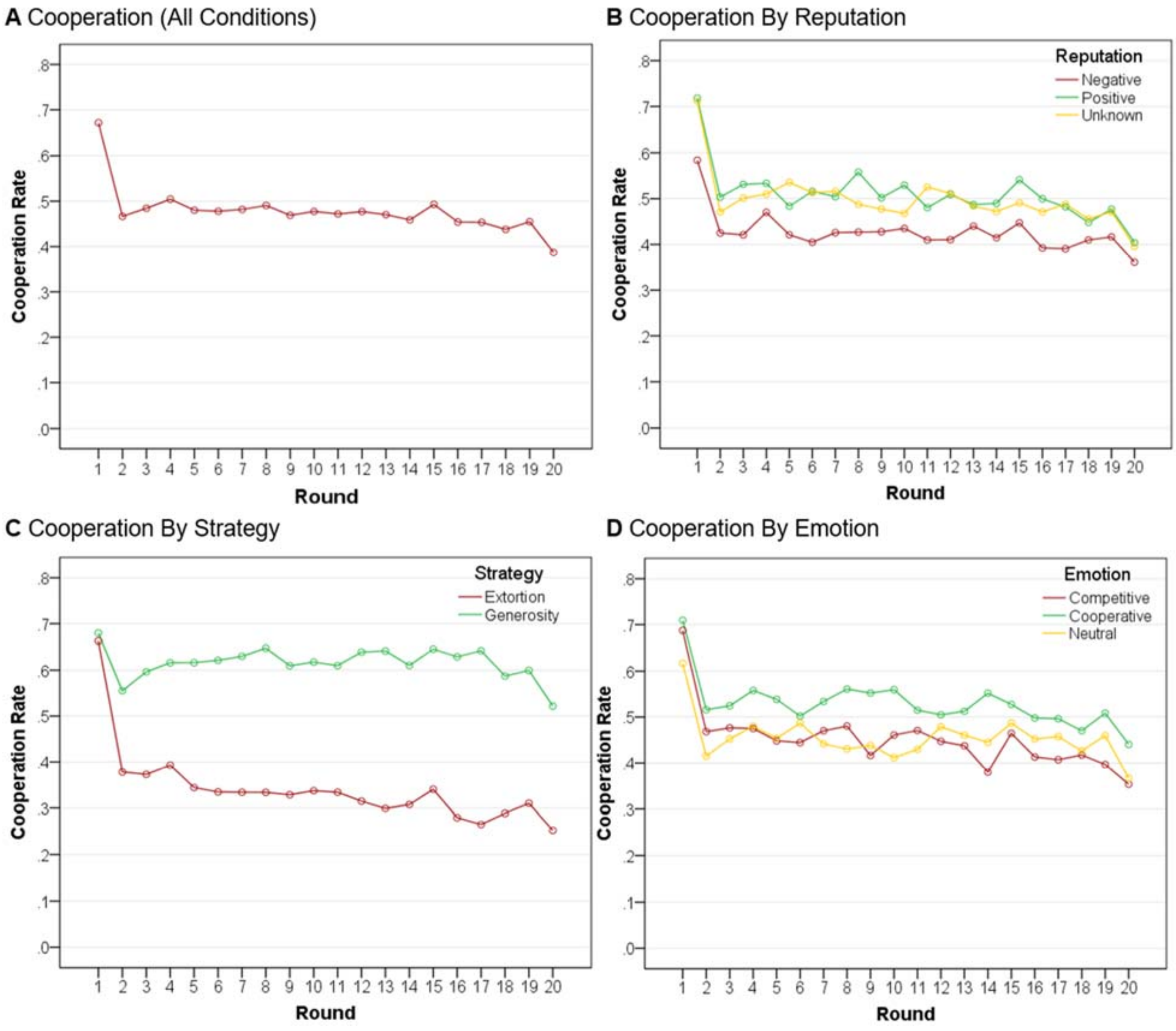

Figure S2. Cooperation per round, Related to Figure 2: (**A**) Collapsed across all conditions, (**B**) Collapsed by reputation, (**C**) Collapsed by strategy, (**D**) Collapsed by emotion expression.

Table S1.

Final reputation and reputation changes for each combination of counterpart's reputation, strategy, and emotion, Related to Figure 2. * Final reputation perception is statistically significantly different than zero. † Reputation change is statistically significantly different than zero.

Reputation	Strategy	Emotion	Final Reputation		Reputation Change	
			Mean	*SD*	*Mean*	*SD*
Negative	Extortion	Competitive	-17.77 *	5.58	-5.33	5.75
		Neutral	-16.10 *	4.88	1.47	5.02
		Cooperative	-13.51 *	5.31	-2.49	5.47
	Generosity	Competitive	2.37	5.65	17.76 †	5.82
		Neutral	4.23	5.25	30.23 †	5.41
		Cooperative	16.19 *	5.80	26.75 †	5.98
Unknown	Extortion	Competitive	-17.89 *	5.14	-25.35 †	5.29
		Neutral	-16.59 *	5.58	-27.49 †	5.75
		Cooperative	-5.15	4.83	-11.19 †	4.98
	Generosity	Competitive	24.85 *	5.51	18.03 †	5.67
		Neutral	29.63 *	6.16	19.56 †	6.34
		Cooperative	34.51 *	5.44	28.73 †	5.60
Positive	Extortion	Competitive	-12.53 *	5.08	-52.98 †	5.23
		Neutral	5.50	5.97	-27.76 †	6.15
		Cooperative	13.81 *	6.26	-21.77 †	6.45
	Generosity	Competitive	33.68 *	5.97	-3.76	6.15
		Neutral	36.53 *	6.36	-5.60	6.55
		Cooperative	36.29 *	5.97	-5.59	6.15
